# The complete mitochondrial genome of a basal teleost, the Asian arowana (*Scleropages formosus, Osteoglossidae*)

**DOI:** 10.1186/1471-2164-7-242

**Published:** 2006-09-21

**Authors:** Gen Hua Yue, Woei Chang Liew, Laszlo Orban

**Affiliations:** 1 Reproductive Genomics Group, Temasek Life Sciences Laboratory, Singapore; 2 Department of Biological Sciences, The National University of Singapore, Singapore; 3 Molecular Population Genetics Group, Temasek Life Sciences Laboratory, 1 Research Link, NUS, Singapore 117604, Singapore

## Abstract

**Background:**

Mitochondrial DNA-derived sequences have become popular markers for evolutionary studies, as their comparison may yield significant insights into the evolution of both the organisms and their genomes. From the more than 24,000 teleost species, only 254 complete mtDNA sequences are available (GenBank status on 06 Sep 2006). In this paper, we report the complete mitochondrial genome sequence of Asian arowana, a basal bonytongue fish species, which belongs to the order of *Osteoglossiformes*.

**Results:**

The complete mitochondrial genomic sequence (mtDNA) of Asian arowana (*Scleropages formosus*) was determined by using shotgun sequencing method. The length of Asian arowana mtDNA is ca. 16,650 bp (its variation is due to polymorphic repeats in the control region), containing 13 protein-coding genes, 22 *tRNA *and 2 *rRNA *genes. Twelve of the thirteen protein coding genes were found to be encoded by the heavy strand in the order typically observed for vertebrate mitochondrial genomes, whereas only *nad6 *was located on the light strand. An interesting feature of Asian arowana mitogenome is that two different repeat arrays were identified in the control region: a 37 bp tandem repeat at the 5' end and an AT-type dinucleotide microsatellite at the 3' end. Both repeats show polymorphism among the six individuals tested; moreover the former one is present in the mitochondrial genomes of several other teleost groups. The TACAT motif described earlier only from mammals and lungfish was found in the tandem repeat of several osteoglossid and eel species. Phylogenetic analysis of fish species representing *Actinopterygii *and *Sarcopterygii *taxa has shown that the Asian arowana is located near the baseline of the teleost tree, confirming its status among the ancestral teleost lineages.

**Conclusion:**

The mitogenome of Asian arowana is very similar to the typical vertebrate mitochondrial genome in terms of gene arrangements, codon usage and base composition. However its control region contains two different types of repeat units at both ends, an interesting feature that to our knowledge has never been reported before for other vertebrate mitochondrial control regions. Phylogenetic analysis using the complete mtDNA sequence of Asian arowana confirmed that it belongs to an ancestral teleost lineage.

## Background

Most animal mitochondrial genomes contain 37 genes, including 13 protein-coding genes, 2 ribosomal RNAs (rRNA) and 22 transfer RNAs (tRNA) necessary for translation of the proteins encoded by the mtDNA [1]. They also possess a major non-coding control region that contains the initial sites for mtDNA replication and mtRNA transcription. The mitochondrial genome generally evolves at elevated rates (5–10 times) compared to single copy nuclear genes, however its gene order often remains unchanged over long periods of evolutionary time, with some exceptions [1]. The genetic code of mitochondrial genomes is more degenerated and thus less constrained than the universal eukaryotic nuclear code [2]. In most animal mitochondrial genomes the genes are distributed on both strands, whereas in some, all genes are transcribed from one strand (e.g. *Tigriopus japonicus*) [3]. Mitochondrial DNA-derived markers have become popular for evolutionary studies, as the data obtained by their analysis may yield significant insights into the evolution of both the organisms and their genomes [1,4].

Teleosts represent the largest vertebrate group with over 24,000 species, accounting for more than the half of all vertebrates. The ancestors of the oldest extant teleost species found on the earth today is believed to have originated from the Mid-triassic, ca. 200 million years before present [5]. Today's teleosts can be classified into 45 orders with a total of 435 families [6]. Over 160 complete fish mitochondrial genomes – representing more than 25 orders – have been reported in the peer-reviewed literatures and more than 70 additional fully sequenced mitochondrial genomes can be retrieved from GenBank (status on February 20, 2006).

The Asian arowana (dragonfish; *Scleropages formosus*, *Osteoglossidae*) belongs to the order *Osteoglossiformes*, one of the ancestral teleost clades with extant representatives restricted to freshwater habitats [6]. It is one of the most expensive ornamental fish species in the world. The Asian arowana is listed by the Convention on International Trades in Endangered Species of Wild Fauna and Flora (CITES) as a "highly endangered" species, therefore a special permit is required for farms dealing with its culture [7]. There are three basic colour varieties of the Asian arowana: the green, the golden and red with several distinct sub-varieties. They all seem to have originated from different regions of Southeast Asia, which were probably connected through freshwater habitats during the Pleistocene glacial ages (ca. 0.11–1.8 million years ago) [8]. According to currently accepted taxonomy, the *Osteoglossiformes *order encompasses the *Osteoglossoidei *and *Notopteroidei *suborders. The *Osteoglossoidei *suborder contains two families:*Osteoglossidae *and *Pantodontidae*. The *Osteoglossidae *family is made up of seven species: *Scleropages formosus *(range: Southeast Asia), *S. jardinii (*Northern Australia and New Guinea)*, S. leichardti *(Eastern Australia), *Osteoglossum bicirrhosum *(South America), *O. ferreirai *(South America), *Arapaima gigas *(South America) and *Heterotis niloticus *(West Africa and the Nile) [6]. The *Pantodontidae *family contains only one species, the butterfly fish, *Pantodon buchholzi *(West Africa) [6]. Among these eight *Osteoglossoidei *species, mitochondrial genomes have been fully sequenced only from two species: *O. bicirrhosum *and *P. buchholzi *[9]. The sister suborder *Notopteroidei *has three families with 56 species [6], however complete mtDNA sequence is only available for a single species, the goldeneye (*Hiodon alosoides*, *Hiodontoidae*).

Although the Asian arowana is one of the most valuable ornamental teleosts, relatively few scientific papers have been published about the species in peer-reviewed literature. Most of these are classical studies dealing with the taxonomy, and physiology of the species (see e.g. [10-12]), only a recent papers use molecular methods (see e.g. [13-15]). The lack of molecular and genomic information about Asian arowana has hindered the study of its biology. Polymorphic DNA markers are expected to be highly useful tools for the understanding of the biology of Asian arowana.

In this paper we describe the complete mitochondrial genome sequence of Asian arowana that has a unique control region containing two different repeat arrays at its ends. Phylogenetic analysis based on fully sequenced mitogenomes of all four osteoglossid species and sixteen other species from *Euteleostomi *confirmed the position of *Osteoglossoidei *among basal fishes. This mitogenomic sequence will be highly useful for the characterization of mtDNA-based polymorphisms, which in turn will provide useful tools for the analysis of parental care of the species.

## Results and discussion

### Gene content and genome organization

The complete mitochondrial genome of Asian arowana was sequenced with shotgun sequencing method (min. 6X, average 9X coverage). Its total size was found to be ca. 16,651 bp [GenBank:DQ023143]. Except the mitochondrial control region the size of Asian arowana mitochondrial genome was found to be similar to that of silver arowana, butterfly fish and goldeneye [9] [see Additional file 1 for the exact sizes]. The GC content of Asian arowana mitochondrial genome was 46.1%, the highest among mitochondrial genomes of all *Osteoglossiformes *available in Genbank (silver arowana – 43%, butterfly fish – 39% and goldeneye – 42%).

On the whole, the structure of the Asian arowana mitochondrial genome is very similar to that of silver arowana, butterfly fish and bichir [see Additional file 2]. The number and order of genes in the Asian arowana mitogenome [see Additional file 3] were found to be the same as common vertebrate form [1]. It contains 24 RNA and 13 protein-coding genes: 7 subunits of the NADH ubiquinone oxidoreductase complex (*nad1-6 and nad4L*), 3 subunits of the cytochrome c oxidase complex (*cox1-3*), a single subunit of the ubiquinol cytochrome c oxidoreductase complex (*cob*), 2 subunits of ATPase (*atp6 and atp6*), 2 ribosomal RNA (*rrnL and rrnS*) and 22 transfer RNA (*trn*) genes. The non-coding control regions situated between the *trnP *and *trnF *genes contain the heavy strand origin of replication (O_H_). A smaller control region containing the putative light strand origin of replication (O_L_) was found between *trnW *and *trnY *genes.

Eleven potential overlaps between genes have been observed in the Asian arowana mitogenome. The longest one (10 bp) involving the two ATPase genes appears to be common in most vertebrate mitochondrial genome, and its size in fish (7–10 bp [16]) is smaller than that in mammals (40–46 bp; [2]). The second largest overlap is 7 bp long, (between *nad4 *and *nad4L *genes), whereas the remaining nine were in the size range of 1–5 bp.

### Mitochondrial control region

**Figure 1:**
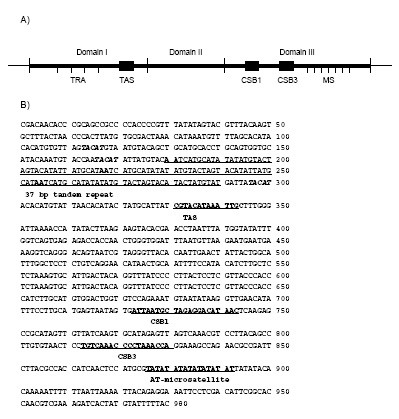
**The schematic diagram and full sequence of the Asian arowana heavy strand control region shows the presence of two repeats**. Panel **A**: Schematic diagram of Asian arowana mitochondrial heavy strand control region. Labels: TRA – tandem repeat array; TAS – termination-associated sequence; CSB – conserved sequence block; MS – microsatellite. Panel **B**: The nucleotide sequence. Positions of the TACAT motif, 37-bp tandem repeat, termination associated sequences, conserved sequence blocks and the AT-microsatellite are labeled with bold.

The Asian arowana mtDNA's heavy strand control region, also known as D-loop, contains O_H _and is ca 980 bp long. Similar to typical vertebrate mitogenomes, this non-coding region can be divided into three different domains [17,18] (Figure 1A). Domain I which is 400 bp long, consists of a termination associated sequence (TAS: TACATAAATTG) [19] and several copies of a previously described conserved palindromic motif without any known function [20]. A 37 bp tandem repeat array, suggested to be involved in the regulation of mitochondrial genome replication by forming a thermostable "hairpin" [21], was also found in this domain (see next section for details). Domain II – commonly known as the central conserved block – covering the 401–641 bp stretch in the control region, showed high similarity to domain II of rainbow trout [22] and sturgeon [21] (data not shown). In domain III, a TA-dinucleotide microsatellite repeat was present in all the six individuals from which the control region was sequenced. Two conserved sequence blocks (CSB1; 724–742 bp and CSB3; 813–839 bp) found in this domain showed high similarity to CSBs detected earlier in other species [23] whereas CSB2 described earlier in teleosts [24] was not found.

A smaller control region (34 bp) for O_L _exhibited high sequence similarity to the corresponding region in silver arowana, bichir and butterfly fish (data not shown). The AT content of Asian arowana O_L _which was 35.3%, is similar to that of butterfly fish but higher than in silver arowana (31.4%) and much lower than in bichir (44%). The secondary structure of O_L _was suggested earlier to regulate light strand replication [25]. In Asian arowana this secondary structure consists of a perfect 9 bp stem (CCTCCCGCC/GGAGGGCGG) and loop structure. Despite the fact that the control region is the most variable region in animal mtDNAs, most part of the stem (TCCCGCC and AGGCGGA) was found to be conserved in the mitogenomic O_L _of several fish species (including the Asian arowana) and even mammalian ones [26].

### Repeats in the heavy strand control region

The mtDNA of all six Asian arowana individuals tested possess a heteroplasmic tandem repeat array in domain I (Figure 1B). The tandem repeat arrays in the six individual fishes sequenced contained 3 to 6 repeat units, resulting in variable length of the heavy strand control region (976 to 1094 bp long). A partial repeat unit could also be found at the beginning and at the end of the array indicating that it might have been formed by replication slippage [21,27].

**Figure 2:**
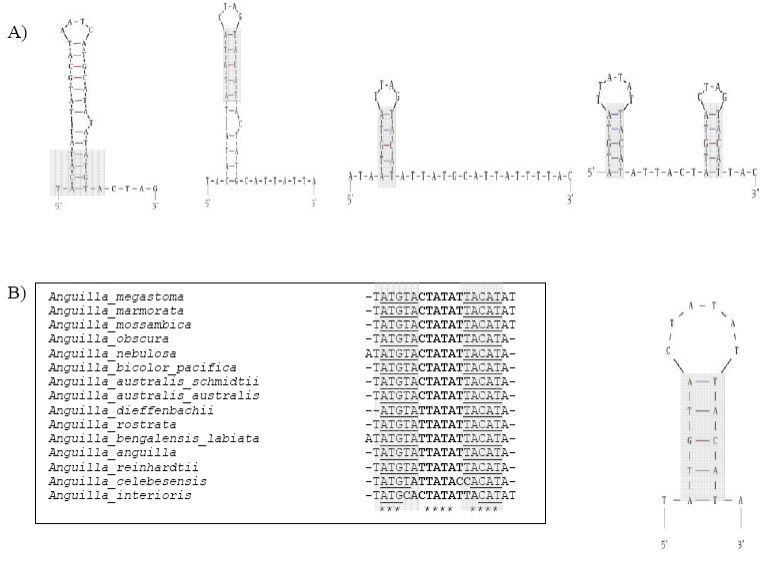
**A conserved motif capable of forming a hairpin is present in the mtDNA of several osteoglossid and eel species**. Mfold deduced hairpin structure from a repeat unit within the tandem repeat array located in heavy strand control region. Shaded region is the conserved motif TACAT/ATGTA. Panel **A**: Hairpin structures of the members of *Osteoglossiformes *superorder. From left: Asian arowana, silver arowana, butterfly fish and goldeneye. Panel **B**: Alignment of a repeat unit sequence from tandem repeat array of various *Anguilliformes *superorder members' mitochondrial heavy strand control region. A hairpin structure of *Anguillia australis australis *constructed using Mfold.

The tandem repeat units were highly similar with only a few base substitutions (Figure 1B). Each repeat unit in the array was 37 bp long (TACATATTATGCATAATCATGCATATATATGTACTAG). The conserved motif TACAT (previously described only in mammals [28] and lungfish [29]) and its complement ATGTA, were both located in the stem region providing the theoretical ability of forming a stable hairpin loop (Figure 2A). Our investigation of the other three members of *Osteoglossiformes *with fully sequenced mitogenome (i.e. silver arowana, butterfly fish and goldeneye) has shown that this conserved motif could also be found in a similar arrangement in their heavy strand control region (Figure 2A). Further investigation revealed that the two motifs could also be found in the heavy strand control region of several eel species (*Anguilliformes*) (Figure 2B). The conservation of this motif across various vertebrate taxa suggests that it serves an important role in the mitochondrial heavy strand control region. An extensive search of the literature database showed that since it was reported more than a decade ago, no extensive study was published to investigate its function. Based on the position of the motif, we speculate that it might be required for the formation of a thermostable hairpin involved in replication of the tandem repeat array. We also cannot rule out the possibility of the motif being binding sites for proteins involved in replication.

Another type of repeat – a TA-type dinucleotide microsatellite – was present at the opposite end of the heavy strand control region in domain III (Figure 1B). The number of TA core units ranged from 8 to 10 in the six individuals sequenced. Although both tandem repeats alone (e.g [30-33]) or microsatellites in the tandem repeat array [34] has been reported earlier in the heavy strand control regions of some species, to our knowledge no one has reported the existence of both types of repeats on the same heavy strand control region.

### Protein-coding genes

The start codon usage in the Asian arowana mitogenome was found to be the same as that of zebrafish [16]. All but one of the 13 protein coding genes began with the orthodox ATG start codon, only *cox1 *used GTG start codon [see Additional file 2]. Ten genes ended in a complete termination codon, either TAA, TAG or AGA. The remaining three genes (*cox2*, *nad4 *and *cob*) did not possess a complete stop codon, but did show a terminal T. This condition is known to be common to vertebrate mitochondrial genomes whereby post-transcriptional polyadenylation provides the two adenosine residues required for generating the TAA stop codon [35].

The total nucleotide length for the 13 coding genes was found to be 11,403 bp, shorter than that of silver arowana and butterfly fish, but longer than that of bichir [see Additional file 2 for the exact sizes]. The coding sequences in Asian arowana consisted of 28.0% A, 25.1% T, 14.8% G and 32.1% C bases. The corresponding ranges for silver arowana, butterfly fish and bichir were 29.2–30.4% (A), 27.9–31.3% (T), 13.4–14.2% (G), and 24.3–28.8% (C). These data further support the observations: i) the GC content of Asian arowana mitogenome is higher than that of other teleost, including other known *Osteoglossoidei *species; and ii) the frequency of G is the lowest among the four bases in fish mitochondrial genomes [2].

Comparison of amino acid sequences for the 13 proteins among Asian arowana, silver arowana, butterfly fish and bichir confirmed the closer taxonomic relatedness of Asian arowana to silver arowana, than to butterfly fish or bichir (Table 1). In agreement with others' data [2,36], *cox1 *was the most conserved gene and *atp8 *was the most variable.

**Figure 3:**
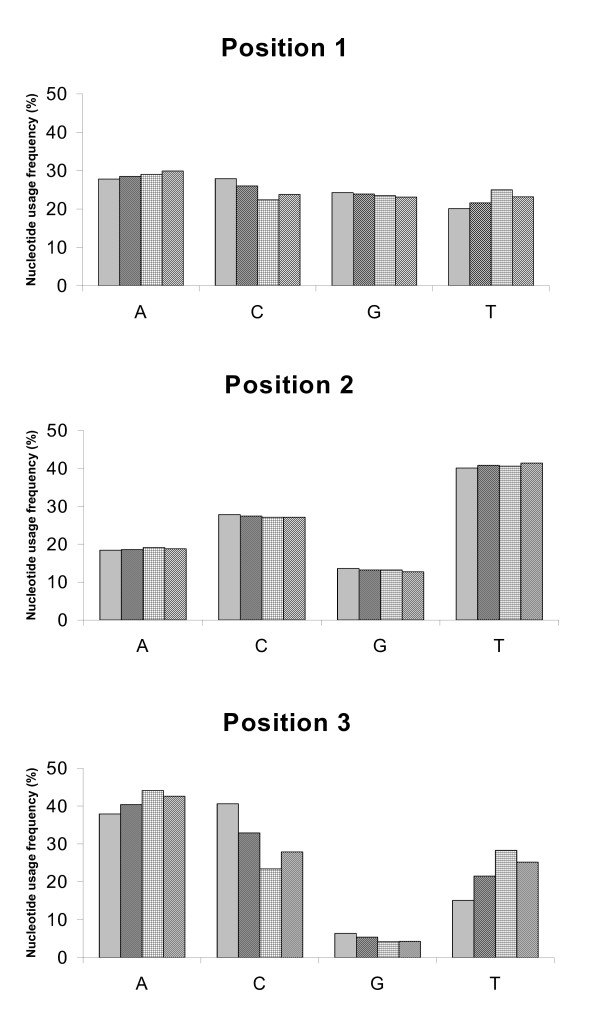
**Nucleotide usage frequency of three osteoglossids compared to that of the bichir**. Frequency of nucleotide usage according to codon position for all protein-coding genes. Order of bars from the left: Asian arowana (), silver arowana (), butterfly fish () and bichir ().

The pattern of codon usage in Asian arowana mtDNA was also studied. The most frequently used amino acids were leucine (16.9%), followed by threonine (8.9%), alanine (8.4%) and glycine (7.8%) [see Additional file 4]. At the third codon position, the order of nucleotide usage frequency was C > A > T > G (Figure 3), the same order was described earlier for the mitochondrial genome of Japanese fugu [37]. The order was somewhat different in the silver arowana, butterfly fish and bichir, where A became the most frequently used base in the third codon position, albeit the frequency of G remained the lowest (Figure 3).

**Figure 4:**
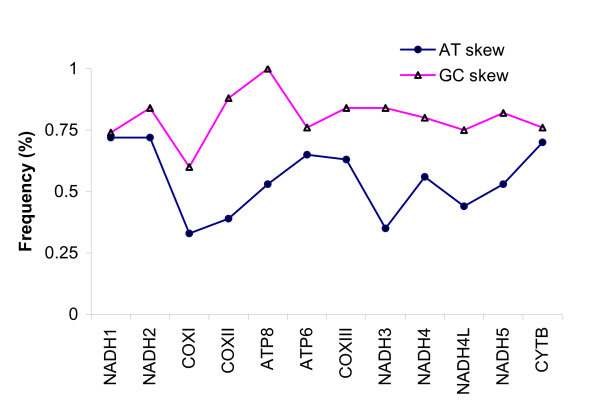
**The GC and AT skew for mitochondrial protein-coding genes in Asian arowana mtDNA**. Graphical representation of absolute values is shown. Genes are ordered according to their position in the mitochondrial genome.

For amino acids with fourfold degenerate third codon position, codons ending with A were the most frequent (42.7%), followed by C (36.5%), T (14.1%) and G (6.2%). Genes located on the heavy strand showed a typical native GC and positive AT skew (Figure 4), whereas the *nad6 *gene located on the light strand displayed an opposite pattern. With regard to the absolute value, the GC skew was always higher than the AT skew: the former ranged from 0.60 to 1, whereas the latter ranged from 0.33 to 0.72 (Figure 4). Similar patterns were also seen in silver arowana, butterfly fish and bichir (data not shown). The GC and AT skews in Asian arowana were not correlated (R = 0.094, P > 0.05).

### Transfer RNA genes

The twenty-two tRNA genes typical of vertebrate mitochondrial genomes have all been detected in the Asian arowana mitogenome. All tRNA genes possessed anticodons that match the vertebrate mitochondrial genetic code. The length of tRNA genes ranged from 67 bp to 74 bp [see Additional file 3] with a total length of 1,550 bp, similar to silver arowana and bichir, but shorter than that in butterfly fish [see Additional file 2]. The inferred secondary structure of the 22 tRNA genes had several uniform features: 7 bp in the aminoacyl stem, 5 bp in the TφC and anticodon stem, 4 bp in the DHU stem and 7 bp in the anticodon loop. A "U" residue before the anticodon was found in 19 of the 22 tRNA, whereas a purine was detected in the position immediately 3' to the anticodon. In the stem regions, there were several non-complementary pairings, mainly A-C type. A similar structure has been found in the silver arowana, whereas different kinds of non-standard base pairings have also been described in other fish species [2]. The original sequences and the secondary structure of the tRNA genes were quite different in genetically distant related species.

### Ribosomal RNA genes

Like the mitochondrial genome of other fishes, the Asian arowana mitogenome was found to possess two ribosomal RNA (rRNA) genes, a small rRNA gene (*rrnS*) and a large rRNA gene (*rrnL*), the two being separated by *trnV *[see Additional file 3]. The length of *rrnS *and *rrnL *are 956 and 1,698 bp, respectively [see Additional file 3]. These sizes are similar to those in the other three species used for comparison [see Additional file 2]. Substitution rates of the two rRNAs among Asian arowana, butterfly fish and bichir were lower than those of protein coding genes. Secondary structures found in the four species seemed to be conserved across large evolutionary distances, as described earlier for teleosts [2].

### Phylogenetic analysis of the *Osteoglossomorpha *superorder

Several studies have been published recently on the phylogeny of *Osteoglossoidei *suborder using morphological data [11], partial mitochondrial sequences [38], a few nuclear genes [39] or the combination of the latter two [40]. On the other hand, there is only a single study that analysed the phylogenetic relationship of the osteoglossids based on all genes present in the mitochondrial genomes [41] but the Asian arowana was not included as its complete mitogenomic sequence was not available.

To determine whether the addition of the complete Asian arowana mitogenome causes any difference in the evolutionary position of the *Osteoglossomorpha *from the cladograms produced earlier [11,38-40], we used the complete Asian arowana mitogenome sequence obtained in this study and other osteoglossids' complete mitogenome sequences to carry out phylogenetic analysis. Beside the osteoglossid species our analysis also included mtDNA from three fish species of ancestral lineages: four members of the *Chondrostei *taxon and representatives for both the *Elopomorpha *and *Clupeocephala *taxa (for complete list of species used, refer to Additional file 1). Using both nucleotide and amino acid sequences of different kinds of mitochondrial genes (see Materials and Methods for details) the systematic arrangements were reconstructed as monophyletic which is in agreement with the relationship tree of basal Actinopterygians produced by Inoue and colleagues [41].

**Figure 5:**
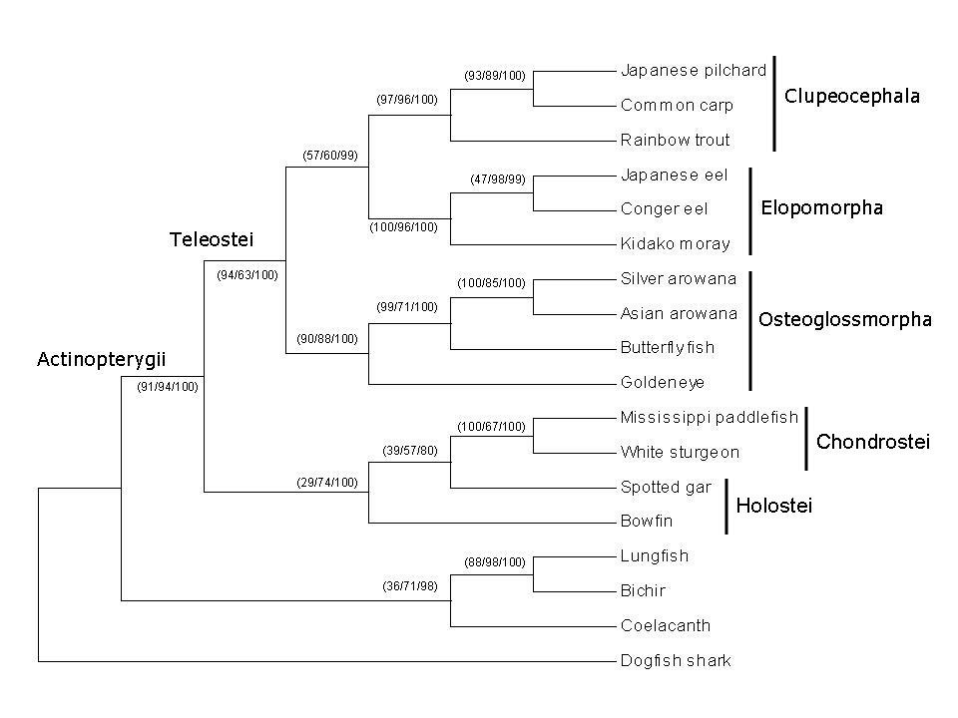
**Phylogenetic analysis of osteoglossids and other teleosts by using concatenated mitochondrial protein-coding genes**. The data sets consist of a total of 3,675 amino acid positions concatenated from 12 protein sequences for each species. The phylogenetic relationship of Asian arowana with respect to representatives from *Actinopterygii *and *Sarcopterygii *taxa using dogfish shark as outgroup was performed by maximum parsimony (MP), maximum likelihood (ML) and Bayesian inferences (BI) methods. Tree topology produced by the different methods was similar. Bootstrap values are in parentheses and in MP/ML/BI order.

Phylogenetic trees constructed with the various data sets using three different methods (i.e. MP, BI and ML) showed little variations within the data set, indicating that variation mainly originated from the type of data and not the methods used (data not shown). On all trees the Asian arowana was clustered into one group with the silver arowana, butterfly fish and goldeneye (all three from the *Osteoglossomorpha *superorder) with a high bootstrap support value. However, within *Teleoste*i taxon the position of *Osteoglossomorpha *clade varied in the trees generated using concatenated protein-coding cum tRNA nucleotide sequences and concatenated protein-coding cum tRNA cum rRNA nucleotide sequences data sets. On the other hand, trees generated using concatenated protein-coding nucleotide sequences and concatenated amino acid sequences data sets consistently placed the *Osteoglossomorpha *clade at the basal level (Figure 5). This is in agreement with trees constructed earlier by others using various molecular [9,38-41] and morphological data [11]. In addition the proximity of *Osteoglossomorpha *clade to that of basal teleost clades in our study further supports the position of osteoglossids among the early branches of living teleosts' stem lineages (see e.g. [41])

The placement of goldeneye and butterfly fish was different in osteoglossid cladograms produced earlier on the basis of morphological data [41-46]. While most publications predicted that during the evolution of osteoglossids the ancestor of goldeneye split off earlier from the arowanas, than from the butterfly fish [42-45]. One study proposed exactly the opposite [46]. Our cladogram based on full mtDNA sequences similarly to the data from [41] from four osteoglossids supports the former situation (Figure 5).

Since goldeneye is the only complete mtDNA sequence reported for *Notopteroidei *suborder, additional full mitogenomic sequences from this taxonomic group will have to be obtained for a more detailed analysis.

## Conclusion

Although the length, gene content and gene order of the mitochondrial genome of Asian arowana is similar to those of other teleost and vertebrate mitochondrial genomes, it exhibits a number of interesting characteristics. Among them the most interesting is the presence of two different kinds of polymorphic repeat sequences at the opposite ends of the mitochondrial control region. These repeats could be potentially useful for the analysis of genetic diversity of populations, as well as phylogenetic and phylogeographic studies of the Asian arowana and possibly other members of *Osteoglossoidae *family. The complete mitogenome of Asian arowana provides an additional important dataset for the study of osteoglossids and other basal fish species.

## Materials and methods

### Sample collection and preparation

Six adult Asian arowana individuals (two green, two red and two golden variety) were obtained from a fish farm in Singapore. A small piece of fin clip (ca. 0.5 cm^3^) was collected from every individual and kept in absolute ethanol at 4°C. Whole genomic DNA including nuclear and mitochondrial DNA was isolated using a quick method developed previously in our laboratory [47].

### PCR amplifications

Two pairs of primers (Dmt-A1/B1 and Dmt-A2/B2, see Additional file 5) were designed from nucleotide sequences of *nad2 *gene [GenBank:AB035222] and *cob *gene [GenBank:AB035234] deposited in Genbank. Long distance PCRs were carried out using Expand Long Template PCR System (Roche) to amplify 2 overlapping fragments of the complete mitochondrial genome. Each 50 μl reaction volume contained 1 × PCR buffer 2 (Roche) with 2.0 mM MgCl_2_, 200 nM of each primer, 400 μM dNTP, 50 ng genomic DNA of one green Asian arowana and 3 U Taq polymerase mix (Roche). The following PCR program was used: 10 cycles of 94°C for 10 sec, 63°C for 30 sec and 68°C for 8 min then 19 cycles of 94°C for 10 sec, 63°C for 30 sec and 68°C for 5 min with an addition of 20 sec/cycle, as well as a final extension at 68°C for 10 min. Primer pair Dmt-A1B1 amplified a fragment of ca. 7.3 kb and primer pair Dmt-A2B2 produced ca. 11.5 kb product. The two fragments overlapped at both ends by a total of ca. 2 kb.

### Shotgun sequencing and assembly

PCR products (25 μl) were sonicated using Branson digital sonicator model 450 (Branson) at 20% power for 4 seconds to generate DNA fragments suitable for cloning into a plasmid (400 bp to 2 kb). Sonicated PCR products were treated with T4 DNA polymerase, Klenow DNA polymerase and T4 polynucleotide kinase (all from Stratagene) according to manufacturer's protocol to blunt and phosphorylate the ends. Treated DNA fragments were electrophoresed through a 1% low melting agarose gel (Bio-Rad). Fragments between 500 bp and 1.5 kb size were cut out from the gel and cleaned using self-made glassmilk as described previously [13]. The isolated DNA fragments were ligated into pBluescript KS (-) (Stratagene) vector pre-digested with *SmaI *(Stratagene). Nucleotide sequencing of the cloned inserts was conducted by using BigDye assay kit version 3.1 (Applied Biosystems) and M13F/M13R sequencing primer [see Additional file 5) as described previously [13]. One hundred and forty-four clones with average insert length of 1 kb were sequenced; representing at least 6 times coverage of each position in the complete mitochondrial genome. Flanking vector sequences were clipped automatically by using commercially available software Sequencher (GeneCodes) with manual correction. Sequences were assembled by using the same software. The complete sequence of Asian arowana mitochondrial genome was deposited in NCBI's Genbank [GenBank:DQ023143].

### Identification of genes

*tRNA *genes were identified as described by Lowe and Eddy [48] with a cove cutoff score of 0.1. Protein and ribosome RNA genes were identified by sequence similarity to their orthologs from other mitochondrial genomes. The 5' ends of protein-coding genes were inferred to be at the first legitimate in-frame start codon (ATN, GTG, TTG and GTT) [49] that did not overlap with the preceding gene, except with an upstream tRNA gene and was limited to the most 3' nucleotide of the tRNA. Protein gene termini were inferred to be at the first in-frame stop codon (TAA, TAG, AGA and AGG). In some genes a T or TA nucleotides adjacent to the beginning of a downstream gene was designated as the truncated codon and assumed to be completed by polyadenylation after transcript cleavage [35].

### Base composition and codon usage

Editseq and GeneQuest software (both from Dnastar) were used to analyze base composition and codon usage. Compositional skew, which indicates compositional difference between the two strands, was calculated using the following formula proposed by Perna and Kocher [50]:

GC skew = (G-C)/(G+C)

and

AT skew = (A-T)/(A+T)

,where C, G, A, and T are the frequencies of the four bases at third codon position of the eight fourfold degenerate codon families.

### Characterization of the AT microsatellite in the heavy strand control region

A pair of primers (Dmt-MS-A/B, see Additional file 5) was designed using PrimerSelect (DnaStar) to flank the microsatellite site in heavy strand control region. One of the primers was labeled with a fluorescent dye 6FAM at the 5' end. The PCR mastermix consisted of 1 × PCR buffer with 1.5 mM MgCl_2 _(Finnzyme), 200 nM primer, 400 μM dNTP, 40 ng genomic DNA, and 1 U DyNAzyme polymerase (Finnzymes). The amplification was performed in a PTC-100 PCR machine (MJ Research) using the following program: 94°C for 2 min, 34 cycles of 94°C for 30 sec, 55°C for 30 sec and 72°C for 30 sec followed by a final extension at 72°C for 5 minutes. PCR products were separated on an ABI 377 DNA sequencer (Applied Biosystems) as described previously [13]. All six individuals were genotyped to detect possible polymorphism.

### Characterization of the long tandem repeat in the heavy strand control region

For characterization of long tandem repeat in the heavy strand control region, we designed one pair of primers (Dmt-LA/LB, see Additional file 5) flanking the control region using PrimerSelect (DnaStar). Complete heavy strand control region was amplified from total genomic DNA of the six individuals used earlier for microsatellite genotyping under the following PCR conditions: 94°C for 3 min, 30 cycles of 94°C for 30 sec, 55°C for 30 sec and 72°C for 1 min, followed by a final extension at 72°C for 5 minutes. PCR products were cleaned using home-made glassmilk [47] before cloning into pGEM-T cloning vector (Promega). Clones were sequenced from both directions using M13F and M13R sequencing primers and BigDye Assay Kit version 3.1 (Applied Biosystems). Forward and reverse sequences were assembled by using Sequencher (GeneCodes).

For detailed study of the control region tandem repeat array, various *Osteoglossiformes *species (see Fig 2 for the species used) and *Anguilla *species (see Fig 2 for the species used) sequences were downloaded from NCBI Genbank. A single unit from the various tandem repeat array were then aligned using ClustalX [52]. Hairpin structure of a repeat unit from the tandem repeat array was constructed using Mfold [51].

### Phylogenetic analysis

Phylogenetic analysis was performed using mitochondrial genome of eighteen fish species from representatives of *Actinopterygii *and *Sarcopterygii *taxa – among them all four available full mtDNA from the *Osteoglossiformes *superorder – were used [see Additional file 1]. A shark species, called spiny dogfish (*Squalus acanthias*, *Squaliformes, Chondrichthyes*) was used as outgroup. Four different data sets were analysed: i) concatenated protein-coding, tRNA and rRNA nucleotide sequences; ii) concatenated protein-coding and tRNA nucleotide sequences; iii) concatenated protein-coding nucleotide sequences and iv) concatenated protein-coding amino acid sequences. Amino acid sequences were aligned using ClustalX [52] then its nucleotide sequences were aligned with references to the amino acid sequences alignment using CodonAlign 2.0 [53]) and further edited manually. The full sequence of *nad6 *encoded by the L strand was excluded from the analysis, due to the deviating nucleotide and amino acid composition of this gene as compared to those encoded by the H strand. Third codons of the 12 heavy strand encoded protein-coding genes were excluded from the analysis together with loops of tRNA. Each of the four datasets were analyzed by maximum parsimony (MP) method in MEGA version 3.1 [54], Bayesian inference (BI) method using MRBAYES 3.1.2 [55,56] and maximum likelihood (ML) using Tree-Puzzle version 5.2 [57] for amino acids data set and TreeFinder version of May 2006 [58] for nucleotide data sets.

For MP analysis 10 random additions were done using the close-neighbour-interchange option with search level 1. Bootstrap analysis with 1000 replicates was conducted.

To find the best model for the nucleotides and amino acid data sets, we applied Modeltest version 3.7 [59] and Prottest version 1.3 [60] respectively. The best-fit model was GTR + I + G for all nucleotide data sets and mtRev + I + G for the amino acid data set. BI method was performed for 10^6 ^generations using nucleotide data sets and 5 × 10^5 ^generations for the amino acid sequences data set. The first 25% of samples were discarded as burn-in.

Quartet-based ML analysis for amino acid data set was performed using TreePuzzle [57]. 1000 steps were performed and the mtRev24 substitution model was used. For parameter estimation, quartet sampling and NJ tree option was chosen. For ML analysis of nucleotides data sets, the program TreeFinder [58] was used. GTR substitution model was used and bootstrap analysis was performed with 1000 replicates.

## Abbreviations

*cox1-3 *– cytochrome oxidase subunits I, II, and III; *cob *– cytochrome oxidase b; *atp6 *and atp8 – ATP synthase subunits 6 and 8; *nad1-6 *and *nad4L *– NADH dehydrogenase subunits 1-6 and 4L; *rrnS *and *rrnL *– Small and large ribosomal RNA; *trn *– transfer RNA; O_H _– Heavy strand origin of replication; O_L _– Light strand origin of replication. MP – maximum parsimony; ML – maximum likelihood; BI – Bayesian Inference.

## Authors' contributions

GHY designed and conducted the experiments, performed most of the data analyses, and drafted the manuscript. WCL has performed the comparative analysis of the 37 bp tandem repeat in teleost mtDNAs and the phylogenetic comparison of the mitogenomes. LO has initiated and led the research project on comparative analysis of Osteoglossids genomes, helped with the experimental design and finalized the manuscript. All authors read and approved the final manuscript.

## Supplementary Material

Additional file 1 Table of complete mtDNAs used for the phylogenetic comparison. This table provides details of the fish species used in this study for phylogenetic analysis. Click here for file

Additional file 2 Mitogenome of representative fish species. This table compares the genome structure of mtDNA from five representative fish species. Click here for file

Additional file 3 The inferred organization of the Asian arowana mitochondrial genome. This table provides details on the Asian arowana mitochondrial genes. The information provided are: i) position; ii) size in bp; iii) start and stop codon used; and iv) length of 5' spacer (bp). Click here for file

Additional file 4 Codon usage of the Asian arowana mtDNA. This table provides data on the frequency of the various codons used in the Asian arowana mitogenome. Click here for file

Additional file 5 Primers used for PCR amplifications. This table provides nucleotide sequence of the primers used in this study. Click here for file

## Figures and Tables

**Table 1:** Comparison of protein lengths and similarities among the mitochondrial protein-coding genes of three osteoglossids and bichir

Gene products	Protein length (in amino acids)				Similarity to Asian arowana (%)		
	
	Asian arowana	Silver arowana	Butterfly fish	Bichir	Silver Arowana	Butterfly fish	Bichir
*nad1*	323	323	322	319	92.0	58.7	57.7
*nad2*	347	348	347	345	82.5	74.4	61.7
*cox1*	518	521	546	518	96.3	94.6	90.9
*cox2*	230	230	230	230	93.0	82.6	74.2
*atp8*	55	55	53	55	68.5	52.8	42.6
*atp6*	227	227	228	227	89.9	85.0	72.2
*cox3*	116	116	115	115	93.1	76.5	73.0
*nad3*	98	98	98	98	93.9	82.7	72.5
*nad4L*	262	261	261	263	94.3	90.8	88.1
*nad4*	460	460	460	459	88.0	82.2	72.3
*nad5*	613	614	615	613	86.5	79.2	66.2
*nad6*	172	172	172	167	94.2	72.1	49.7
*cob*	380	381	380	380	91.1	88.2	78.9
