# Supplementary material for: The complete mitochondrial genome of a basal teleost, the Asian arowana (Scleropages formosus, Osteoglossidae)
Source: BMC Genomics. 2006 Sep 21;7:242. doi: 10.1186/1471-2164-7-242 (PMC1592092; doi:10.1186/1471-2164-7-242)
Supplement: Additional file 1 — Table of complete mtDNAs used for the phylogenetic comparison. This table provides details of the fish species used in this study for phylogenetic analysis. [file 1471-2164-7-242-S1.doc]

Table of complete mtDNAs used for the phylogenetic comparison.

| Order | Species | Common name | Size (bp) | GenBank No. |
| --- | --- | --- | --- | --- |
| *Acipenseriformes* | *Acipenser transmontanus* | White sturgeon | 16,692 | AB042837 |
| *Acipenseriformes* | *Polyodon spathula* | Mississippi paddlefish | 16.512 | AP004353 |
| *Amiiformes* | *Amia calva* | Bowfin | 16,210 | AB042952 |
| *Anguilliformes* | *Anguilla japonica* | Japanese eel | 16,685 | AB038556 |
| *Anguilliformes* | *Conger myriaster* | Conger eel | 18,075 | AB038381 |
| *Anguilliformes* | *Gymnothorax kidako* | Kidako eel | 16,579 | AP002976 |
| *Clupeiformes* | *Sardinops melanostictus* | Japanese pilchard | 16,881 | AB032554 |
| *Coelacanthiformes* | *Latimeria chalumnae* | Coelacanth | 16,407 | U82228 |
| *Cypriniformes* | *Cyprinus carpio* | Common carp | 16,575 | X61010 |
| *Lepidosireniformess* | *Protopterus dolloi* | Lungfish | 16,646 | L42813 |
| *Osteoglossiformes* | *Scleropages formosus* | Asian arowana | 16,651 | DQ023143 |
| *Osteoglossiformes* | *Osteoglossum bicirrhosum* bicirrhosum | Silver arowana | 16,006^**^ | AB043025 |
| *Osteoglossiformes* | *Pantodon buchholzi* | Butterfly fish | 15,845^**^ | AB043068 |
| *Osteoglossiformes* | *Hiodon alosoides* | Goldeneye | 16,619 | AP004356 |
| *Polypteriformes* | *Polypterus ornatipinnis* | Bichir | 16,624 | U62532 |
| *Salmoniformes* | *Oncorhynchus mykiss* | Rainbow trout | 16,642 | L29771 |
| *Semionotiformes* | *Lepisosteus oculatus* | Spotted gar | 16,330 | AB042861 |
| *Squaliformes* | *Squalus acanthias* | Spiny dogfish | 16,738 | Y18134 |

** The sequence of control region is incomplete.
