# Supplementary material for: The complete mitochondrial genome of a basal teleost, the Asian arowana (Scleropages formosus, Osteoglossidae)
Source: BMC Genomics. 2006 Sep 21;7:242. doi: 10.1186/1471-2164-7-242 (PMC1592092; doi:10.1186/1471-2164-7-242)
Supplement: Additional file 2 — Mitogenome of representative fish species. This table compares the genome structure of mtDNA from five representative fish species. [file 1471-2164-7-242-S2.doc]

### Mitogenome of representative fish species

| Species | Total length | Protein genes* | rRNA genes | tRNA  genes | Non-coding  sequences | Sequence  overlap | Coding strand |
| --- | --- | --- | --- | --- | --- | --- | --- |
| Asian arowana | 16,651 | 11,403 | 2,654 | 1,552 | 1,043 | -35 | H and L |
| Silver arowana | 16,006 | 11,409 | 2,658 | 1,550 | 395** | -37 | H and L |
| Butterfly fish | 15,845 | 11,481 | 2,637 | 1,554 | 262** | -24 | H and L |
| Goldeneye | 16,619 | 11,412 | 2,657 | 1,559 | 911 | -18 | H and L |
| Bichir | 16,624 | 11,367 | 2,615 | 1,550 | 1102 | -29 | H and L |

* For these protein genes the stop codons were not included.

** The sequence of control region is incomplete.
