# Supplementary material for: The complete mitochondrial genome of a basal teleost, the Asian arowana (Scleropages formosus, Osteoglossidae)
Source: BMC Genomics. 2006 Sep 21;7:242. doi: 10.1186/1471-2164-7-242 (PMC1592092; doi:10.1186/1471-2164-7-242)
Supplement: Additional file 3 — The inferred organization of the Asian arowana mitochondrial genome. This table provides details on the Asian arowana mitochondrial genes. The information provided are: i) position; ii) size in bp; iii) start and stop codon used; and iv) length of 5' spacer (bp). [file 1471-2164-7-242-S3.doc]

### The inferred organization of the Asian arowana mitochondrial genome.

| Gene | Position | Size (bp) | Start | Stop | 5’ space |
| --- | --- | --- | --- | --- | --- |
| O_H_ | 1-980 | 980 |  |  | 0 |
| *trnF* | 981-1050 | 70 |  |  | 0 |
| *rrnS* | 1051-2006 | 956 |  |  | 0 |
| *trnV* | 2007-2078 | 72 |  |  | 0 |
| *rrnL* | 2079-3776 | 1698 |  |  | 0 |
| *trnL* | 3777-3850 | 74 |  |  | 7 |
| *nad1* | 3858-4829 | 972 | ATG | TAA | 3 |
| *trnI* | 4833-4904 | 72 |  |  | -1 |
| *trnQ* | 4974-4904L | 71 |  |  | -1 |
| *trnM* | 4974-5042 | 69 |  |  | 0 |
| *nad2* | 5043-6086 | 1044 | ATG | TAG | -2 |
| *trnW* | 6085-6153 | 69 |  |  | 2 |
| *trnA* | 6224-6156L | 69 |  |  | 1 |
| *trnN* | 6298-6226L | 73 |  |  | 0 |
| O_L_ | 6299-6332 | 34 |  |  | 0 |
| *trnC* | 6399-6333L | 67 |  |  | 0 |
| *trnT* | 6470-6400L | 71 |  |  | 1 |
| *cox1* | 6472-8028 | 1557 | GTG | AGA | -5 |
| *trnS* | 8094-8024L | 71 |  |  | 1 |
| *trnD* | 8096-8165 | 70 |  |  | 4 |
| *cox2* | 8170-8860 | 691 | ATG | T* | 0 |
| *trnK* | 8861-8933 | 73 |  |  | 1 |
| *atp8* | 8935-9102 | 168 | ATG | T* | -10 |
| *atp6* | 9093-9776 | 684 | ATG | TAA | -1 |
| *cox3* | 9776-10564 | 789 | ATG | TAA | -1 |
| *trnG* | 10564-10635 | 72 |  |  | 0 |
| *nad3* | 10636-10986 | 351 | ATG | TAG | -2 |
| *trnR* | 10985-11054 | 70 |  |  | 0 |
| *nad4L* | 11055-11351 | 297 | ATG | TAA | -7 |
| *nad4* | 11345-12725 | 1381 | ATG | T* | 0 |
| *trnH* | 12726-12794 | 69 |  |  | 1 |
| *trnS* | 12863-12796L | 67 |  |  | -1 |
| *trnL* | 12863-12935 | 73 |  |  | 0 |
| *nad5* | 12936-14777 | 1842 | ATG | TAA | -4 |
| *nad6* | 15292-14774L | 519 | ATG | TAG | 0 |
| *trnE* | 15360-15293L | 68 |  |  | 7 |
| *cob* | 15368-16508 | 1141 | ATG | T* | 0 |
| *trnT* | 16509-16581 | 73 |  |  | 1 |
| *trnP* | 16651-16583 | 69 |  |  |  |

T*: Stop codon is completed by the addition of 3’ A residues to the mRNA
