# Supplementary material for: The complete mitochondrial genome of a basal teleost, the Asian arowana (Scleropages formosus, Osteoglossidae)
Source: BMC Genomics. 2006 Sep 21;7:242. doi: 10.1186/1471-2164-7-242 (PMC1592092; doi:10.1186/1471-2164-7-242)
Supplement: Additional file 4 — Codon usage of the Asian arowana mtDNA. This table provides data on the frequency of the various codons used in the Asian arowana mitogenome. [file 1471-2164-7-242-S4.doc]

### Codon usage of the Asian arowana mtDNA.

| Amino acid (anticodon) | Codon  group | Usage of codon ending in | | | | Total | % |
| --- | --- | --- | --- | --- | --- | --- | --- |
|  |  | A | C | G | T |  |  |
| Ala (UGC) | GCN | 83 | 170 | 14 | 51 | 318 | 8.37 |
| Arg (UCG) | CGN | 44 | 20 | 6 | 5 | 75 | 1.97 |
| Asn (GUU) | AAY | - | 95 | - | 19 | 114 | 3.00 |
| Asp (GUC) | GAY | - | 57 | - | 19 | 76 | 2.00 |
| Cys (GCA) | TGY | - | 26 | - | 6 | 32 | 0.84 |
| Gln (UUG) | CAR | 96 | - | 9 | - | 105 | 2.76 |
| Glu (UUC) | GAR | 79 | - | 19 | - | 98 | 2.58 |
| Gly (UCC) | GGN | 78 | 94 | 36 | 32 | 240 | 6.31 |
| His (GUG) | CAY | - | 91 | - | 18 | 109 | 2.87 |
| Ile (GAU) | ATY | - | 191 | - | 104 | 295 | 7.76 |
| Leu (UAG) | CTN | 279 | 158 | 41 | 75 | 553 | 14.55 |
| (UAA) | TTR | 69 | - | 20 | - | 89 | 2.34 |
| Lys (UUU) | AAR | 82 | - | 2 | - | 84 | 2.21 |
| Met (CAU) | ATR | 129 | - | 43 | - | 172 | 4.53 |
| Phe (GAA) | TTY | - | 158 | - | 67 | 225 | 5.92 |
| Pro (UGG) | CCN | 104 | 79 | 8 | 28 | 219 | 5.76 |
| Ser (UGA) | TCN | 77 | 67 | 5 | 35 | 184 | 4.84 |
| UGA | AGN | - | 45 | - | 8 | 53 | 1.39 |
| Thr (UGU) | ACN | 147 | 149 | 6 | 35 | 337 | 8.87 |
| Trp (TGR) | TGR | 99 | - | 19 | - | 118 | 3.10 |
| Tyr (GUA) | TAY | - | 84 | - | 30 | 114 | 3.00 |
| Val (UAC) | GTN | 74 | 61 | 17 | 39 | 191 | 5.02 |
| Total  % |  | 1440  37.89 | 1545  40.65 | 245  6.46 | 571  15.01 | 3801 | 100 |
