# Supplementary material for: The complete mitochondrial genome of a basal teleost, the Asian arowana (Scleropages formosus, Osteoglossidae)
Source: BMC Genomics. 2006 Sep 21;7:242. doi: 10.1186/1471-2164-7-242 (PMC1592092; doi:10.1186/1471-2164-7-242)
Supplement: Additional file 5 — Primers used for PCR amplifications. This table provides nucleotide sequence of the primers used in this study. [file 1471-2164-7-242-S5.doc]

**Primers used for PCR amplifications.**

| Primer name | Sequence (5’-3’) |
| --- | --- |
| Dmt-A1 | CAGCGCCATCCAACATTTCCGCCTGAT |
| Dmt-B1 | CTGGTGTGATGGGGAGGAGCATTAGGGTGAG |
| Dmt-A2 | TTGCCAGCTCACACTGACTCCTAGCCTGAAT |
| Dmt-B2 | GGATGGATGCGATTTGTCCGATGAGGATAAA |
| M13F | GTAAAACGACGGCCAGT |
| M13R | GGAAACAGCTATGACCAT |
| Dmt-MS-A | CCAGAACGCCGATTCTTACGC |
| Dmt-MS-B | TTTGGCTACAGCTGACATAAAAGTCG |
| Dmt-LA | ACTCCTTCCTAGAGCCCTCAGAGA |
| Dmt-LB | GGGCCCATCTTAACAGCTTCAG |
